# Remote, smart telemonitoring of COVID-19 survivors for early detection of deterioration in cardiac health (the PARTMO study)

**DOI:** 10.3389/fmedt.2025.1534097

**Published:** 2025-07-24

**Authors:** Josephine Sau Fan Chow, Nutan Maurya, Susan San Miguel, Rumbidzai Teramayi, Ahilan Parameswaran, Annamarie D’Souza, Gregory Melbourne, Joseph Descallar, Young Juhn, Enoch Chan, Jerome Pong

**Affiliations:** ^1^ South Western Sydney Nursing and Midwifery Research Alliance, South Western Sydney Local Health District, Sydney, NSW, Australia; ^2^ Nursing and Midwifery Research, Ingham Institute for Applied Medical Research, Sydney, NSW, Australia; ^3^ Faculty of Nursing, University of Sydney, Sydney, NSW, Australia; ^4^ Faculty of Medicine, University of New South Wales, Sydney, NSW, Australia; ^5^ Faculty of Medicine, Western Sydney University, Sydney, NSW, Australia; ^6^ Emergency Department, South Western Sydney Local Health District, Sydney, NSW, Australia; ^7^ Administration Department, Wellysis Corporation Ltd., Seoul, Republic of Korea

**Keywords:** arrhythmia, COVID survivors, long COVID, post-COVID clinical symptom, virtual monitoring, model of care

## Abstract

**Introduction:**

This study aims to implement a virtual model of care in the primary healthcare setting, utilising biosensor technologies (S-Patch EX) to remotely monitor and identify clinical signs and symptoms of cardiovascular conditions (mainly arrhythmias) in patients post-COVID-19 infection.

**Methods:**

This open-label, non-randomised, observational study was conducted in patients aged 18 years and above, clinically diagnosed with COVID-19 after June 2021, and those residing within Greater Western Sydney. The study involved two arms: the remote monitoring (intervention) and standard care (control) groups. The intervention group comprised patients who were provided with an S-Patch EX to monitor their electrocardiogram. Data were transmitted in real-time to a mobile phone via Bluetooth technology, and results were generated through artificial intelligence (AI) algorithms. All the data were reviewed for arrhythmia detection and escalated to the participant’s general practitioner (if detected) to determine the appropriate intervention. The control group was used to compare the rate of cardiac arrhythmia detection against the intervention group. The patient’s demographic and longitudinal clinical data were obtained from the electronic medical record system, enabling exploration and comparison of the cohort’s characteristics and outcomes. Descriptive analysis was conducted for categorical variables (frequencies and cross-tabulations) and continuous variables (means, standard deviations, and medians). Depending on the nature of data, the groups were compared using *t*-tests or Chi-square tests. Multivariable Cox regression was used to analyse time to first cardiovascular event post-COVID-19 infection.

**Outcome measures:**

The time to the patient’s first cardiovascular event (mainly arrhythmias) post-COVID-19 infection.

**Results:**

Of 44 patients who provided consent, 40 commenced monitoring. Thirteen patients (32.5%) were detected by the AI algorithms from the S-Patch EX monitoring system to have cardiac arrhythmias, including atrial fibrillation, supraventricular tachycardia, and ventricular tachycardia. Univariate Cox regression demonstrated that arrhythmia was more likely to be detected in the remote monitoring group (13/40, 32.9%) as compared with the standard care group (7/200, 3.5%) [HR = 29.56 (9.95, 87.86), *p* < 0.0001]. Most of the patients were detected with arrhythmia within a 3-month timeframe of monitoring. Twenty-one patients (21/200, 10.5%) from the standard care group visited the emergency department and/or were admitted to the hospital post-COVID-19 infection due to chest pain, shortness of breath/dyspnoea, palpitations, dizziness/light-headedness/presyncope, and nausea. Two patients developed long COVID symptoms (progressive dyspnoea) 2–5 months post-COVID-19 infection.

**Conclusions:**

Considering the risk of developing cardiovascular complications post-COVID-19 infection, regular monitoring, reassessment, and evaluation are recommended as a part of post-COVID-19 management for all patients, including young, healthy, and asymptomatic populations. A randomised interventional study with a larger sample size and longer follow-up period is advised for a better understanding of the cardiovascular impact post-COVID infection.

## 1 Introduction

COVID-19 (COVID) survivors are at increased risk of experiencing a wide range of cardiovascular complications post-COVID infection, regardless of age, race, sex, symptom severity, underlying chronic condition, and care setting, that can last for at least a year and beyond (1–6). A recent systematic review reports a 2.42 times higher risk of any cardiovascular disease (CVD), a 95% higher risk of major adverse CVD events (MACE), a 61% higher risk of arrhythmias, a 71% higher risk of heart failure, a 5 times higher risk of myocarditis, and a 2.49 times higher risk of thrombotic events associated with COVID (7). The long COVID cohort is also reported to experience increased healthcare utilisation for a wide range of adverse outcomes (particularly cardiac) and mortality (8).

The global overall prevalence of long COVID is estimated to be 43%; however, there is a wide variation in estimates, ranging from 9% to 81% (9). In Australia, long COVID (defined as >12 weeks) prevalence ranges from 5% to 10% (10). Since the start of the pandemic and up to 30 September 2022, 1.2% deaths have been due to post-COVID condition (11). There is limited evidence in Australia in relation to the long COVID impact (12–15). Most of the studies conducted comprised hospitalised COVID patients, and the data on post-COVID symptoms were collected via surveys or telephone interviews. A recent study (16) in Victoria reported overrepresentation of impaired global longitudinal strain and left ventricular dimensions in patients with long COVID. However, we did not come across any study monitoring the cardiac health of COVID survivors in the community post-infection.

In recent years, remote sensors, commercial wearable devices, and telemonitoring devices have been used to monitor patients post-COVID infection (17–21). The studies utilising telemonitoring and data from Fitbit devices have shown arrhythmic events during the outpatient monitoring period and modification in the resting heart rate (RHR) for up to 3 months (22) or beyond (23) following symptom onset with substantial intraindividual variability. However, these studies primarily aimed to assess the impact of COVID on an individual's physiology and behaviour (i.e., activity and sleep quantity) post-infection (21–25), not the cardiovascular complications. The follow-up periods were also shorter, e.g., 7 days (19), 10 days (21), and 14 days (24, 25), with only a few follow-ups to 3 months (18, 20) or further (23). There are recommendations that remote patient monitoring should be incorporated into the patient care model to aid in arrhythmia detection as well as monitoring for worsening heart failure in the outpatient setting (26). Yet, there are no clear guidelines or validated models for monitoring cardiac symptoms in COVID survivors in the community to identify deteriorating patients.

To address this critical gap, building on our digital virtual health and artificial intelligence (AI) initiatives, we aimed to evaluate the effectiveness of an innovative digital health model by using wearable biosensor devices and AI to remotely monitor the cardiac health of COVID survivors in the community. The demographic characteristics (age, gender, comorbidities) and clinical outcomes (cardiac arrhythmia) will be collected and compared between the remote monitoring and standard care groups. The study also intended to develop and further refine the communications platform and process of the cardiac care pathway.

### 1.1 Pilot study

A pilot study (TeleCOVID) conducted in 2022 allowed the research team to co-design and implement research activities in real clinical settings, enabling the translation of the findings to the current study in cardiac health screening and monitoring (27). TeleCOVID aimed to test the feasibility of the protocol and communication platform in a real clinical setting and adopt the experience to develop the monitoring model, standard operating procedures, and processes of care pathway for this study. Using the S-Patch wearable biosensor technology, TeleCOVID identified underdetection for over 35% participants (no prior history of arrhythmias) who completed their 3-month follow-up. This allowed early escalation to their treating doctors for further investigations and early interventions. This PARTMO study will expand our proven capability to implement an integrated digital intelligence model of cardiac health intervention and upscale the communication pathway to support digital health in regional settings.

### 1.2 Study aim

This study aimed to implement an innovative and novel virtual model of care in the primary healthcare setting, utilising wearable/portable biosensor technologies (S-Patch EX) to remotely monitor and identify clinical signs and symptoms of cardiovascular conditions (mainly arrhythmias) in patients post-COVID-19 infection and determine whether there was an improvement in cardiac arrhythmia detection in patients in the remote monitoring group compared with the standard care group (without remote monitoring).

## 2 Methods

### 2.1 Study design and intervention

This was an open-label, non-randomised, observational study of an extension of the custom-designed monitoring model of care, capable of recording heart rate and electrocardiogram (ECG). The utilisation of bio-instrumentation technologies (S-Patch) to monitor ECG through an app on a mobile phone allowed the clinicians to view the results via a cloud-based platform, and it was extended to the COVID survivors for a duration of up to 12 months.

#### 2.1.1 S-Patch EX

**Figure 1:**
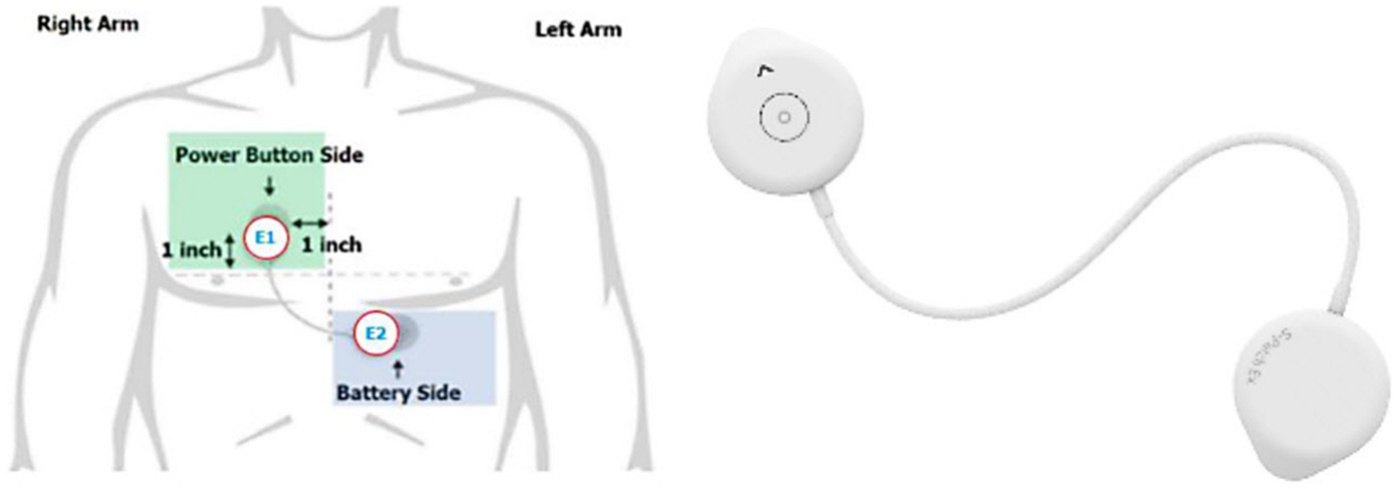
S-Patch EX.

Wellysis, a Samsung SDS spinoff company, developed a bio-processor, a single, compact chip that is capable of measuring galvanic skin response (GSR) and photoplethysmogram (PPG) to sense pulsatile skin blood flow, ECG, skin temperature, and body fat. This bio-processor is built into a device called the “S-Patch EX”, attachable to the chest with two electrode stickers; similar to a ward telemetry monitor that attaches to the chest via five electrode stickers (Figure 1). The bio-processor chip collects the data through the S-Patch device, which is transmitted in real-time to a mobile phone via Bluetooth technology. The data are later transmitted to the cloud (e-server) for initial analysis and reporting, with this data being accessible with secure logon from desktops, tablets, or other mobile devices.

The AI-driven ECG analysis provides an easy-to-read report including clinical information identical to the original Holter test. S-Patch EX is approved for clinical use worldwide and has approval from the Therapeutic Goods Authority (TGA). The S-Patch is validated and provides clinically appropriate ambulatory ECG monitoring (28). It is demonstrated to have higher efficacy in detecting atrial fibrillation (AF) (following cardiologist review) as compared with conventional ward telemetry, allowing patients to be anti-coagulated appropriately for the prevention of further stroke (29).

### 2.2 Setting

The study was conducted in South Western Sydney. The ethical approval for this study was granted by South Western Sydney Local Health District Human Research Ethics Committee (2022/ETH02423).

### 2.3 Participants

The participants in the study were patients aged 18 years and above, clinically diagnosed with COVID-19 (after 22 June 2021) and residing in Greater Western Sydney. The participants were recruited using the South Western Sydney Local Health District COVID-19 Emergency Operation Centre Database. The study contained two arms: the remote monitoring (intervention) group and standard care (control) groups.

#### 2.3.1 Remote monitoring group (intervention)

In the remote monitoring group, the participants were provided with an S-Patch EX device to monitor their electrocardiogram. Included were those who (i) had a smartphone and access to the Internet (Wi-Fi or mobile data) and (ii) provided written informed consent. Excluded were those who had cardiac conditions prior to COVID-19 infection. All the patients were screened and assessed for suitability against the inclusion and exclusion criteria. The identified eligible participants were called as per the telephone script. Interested participants were sent the Participant Information Sheet (PIS) and Consent Form (CF). Depending on the participants’ preferences, the PISCF was sent electronically via the Veeva eConsent platform, email, or post. The participants were given ample time to consider their participation in the study prior to signing the consent form. They could also discuss the study with their doctor or family members before consenting.

#### 2.3.2 Standard care group (control)

The control group was used to compare the rate of cardiac arrhythmia detection against the intervention group. Patients were matched exactly on gender, presence of comorbidity (yes/no), and within 5 years of age. The demographic and longitudinal clinical data of the patient were accessed and extracted via the electronic medical record (eMR), enabling exploration and comparison of the cohort’s characteristics and outcomes.

### 2.4 Study measures

The primary measure was the time to the patient's first CVD event as per the World Health Organization (WHO)'s Global Clinical Platform for COVID case report form, i.e. cardiac arrhythmia (ventricular tachycardia, ventricular fibrillation, long QT, AF, atrial flutter, atrial tachycardia, atrioventricular tachycardia, atrioventricular block of any degree, bradycardia) (30).

### 2.5 Data sources/measurement

#### 2.5.1 Remote monitoring group (intervention)

Once consented, the participants were provided with the S-Patch EX for up to 12 months. Other consumables such as batteries and ECG dots were also provided monthly via mail services. As soon as the participant received the device, they were required to install the S-Patch app onto their mobile phones. The participants were then fitted with the device according to the Product Operations Manual, guided by an experienced researcher via teleconsultation.

The participants were advised to wear the S-Patch and monitor continuously for a period of up to 12 months. However, those doing reasonably well and unable to monitor every day were given an option to monitor during weekends or 2–3 consecutive days per week. In addition, it was recommended to monitor during “bad days” when they experienced any symptom (extreme fatigue, shortness of breath, heart palpitations, chest pain or tightness, problems with memory and concentration, changes to taste and smell, joint and muscle pain). After completion of the study duration, all the participants were provided with a report of their remote monitoring data recorded during the device activation period.

##### 2.5.1.1 Data review

The data were reviewed daily by a study investigator (clinician), with any findings requiring escalation highlighted and forwarded to the medical investigator for further review and advice. The medical investigator had access to the telemetry database to allow for real-time review of ECG. The participants and their GP were then informed of the findings and recommendations, if required.

During monitoring, the participants who were identified with cardiac arrhythmia confirmed by their GP or treating specialist review were separated from the study procedure, as soon as CVD was confirmed. All other participants were otherwise monitored for a period of up to 12 months or until CVD was identified and confirmed.

##### 2.5.1.2 Demographic and clinical variables

The following data were collected from the *COVID-19 Emergency Operation Centre Database and eMR*: gender, age, hospitalisation status, admission date, existing comorbidities, and hospitalisation and/or emergency department visit due to heart issues post-COVID.

Data related to ECG such as QRS complexes, supraventricular ectopy (SVE), and ventricular ectopy (VE) wer obtained from the S-Patch portal.

###### 2.5.1.2.1 QRS complexes

The QRS complex is a combination of the Q wave, R wave, and S wave and represents ventricular depolarisation. In adults, the QRS complex normally lasts 80–100 ms. A QRS duration of >0.12 s is considered abnormal. The QRS complex is an electrical ventricular system and is the most well-known waveform showing electrical activity inside the heart. It is the basis for automatic recognition of heart rate and also serves an access point for classification schemes and ECG data-compression algorithms. The duration, amplitude, and morphology of the QRS complex are useful in diagnosing cardiac arrhythmias (31).

###### 2.5.1.2.2 SVE and VE

Supraventricular and ventricular ectopy are extra, abnormal depolarizations at non-sinus atrial, atrioventricular, or ventricular foci (32). Supraventricular ectopic beats (or premature supraventricular ectopic beats) arise from the top chambers of the heart (atria), and ventricular ectopic beats (or premature ventricular ectopic beats) arise from the lower chambers (ventricles). Early detection of SVE and VE beats is particularly important for preventing dangerous heart diseases (33).

#### 2.5.2 Standard care group (control)

Patients’ demographic (age and gender) and clinical record available on the eMR in relation to COVID and cardiac issues (existing comorbidities, any hospitalisation and/or emergency department visit due to heart issues post-COVID infection, and date of admission to hospital/ED visit) was assessed and obtained for analysis purposes. Patients who had any cardiac conditions (based on the record on the eMR) prior to COVID infection were excluded from the analysis.

### 2.6 Patient and public involvement

The patients or the public were not involved in the design, conduct, reporting, or dissemination plans of this research. Ten healthcare workers were involved in the development of the research methodology. No identifying details or contact information of participants were provided to people other than the authorised research team.

### 2.7 Analysis

A convenience sample of patients was recruited to the intervention arm during the study period. A matched control cohort based on list factors (age, gender, and comorbidities) was identified from the eMR with a 1:5 intervention to control ratio.

Descriptive analysis with frequencies and cross-tabulations for categorical variables and means (standard deviations) and medians (ranges) for continuous variables was conducted. Comparisons between groups were conducted using *t*-tests or Chi-square tests. The univariate Cox proportional hazards model was used to analyse time to patient's first CVD event (mainly arrhythmias). Time to arrhythmias was estimated from date of admission to date of arrhythmia or right censored if there was no event at the end of study period. Multivariable Cox models were considered in the event of confounding and/or unbalance of variables between the groups. Schoenfeld residuals were examined to assess the proportionality assumption with the “cox.zph” command in R. Analyses were performed using SPSS version 26, SAS version 9.4, and R version 4.2. A *P*-value of <0.05 was considered statistically significant.

## 3 Results

### 3.1 Remote monitoring group

**Figure 2:**
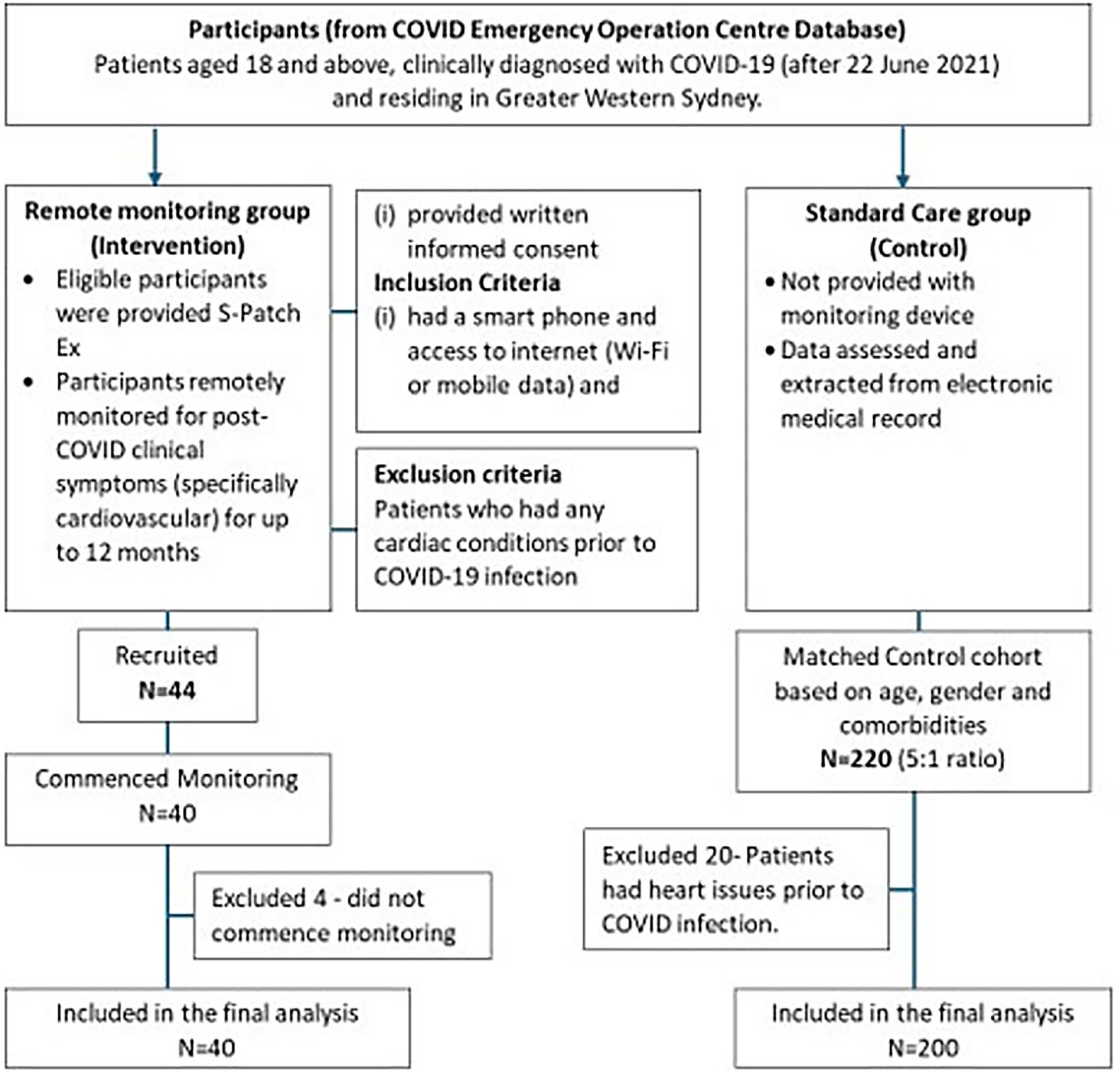
Flowchart describing the study participants’ enrolment process.

Forty-four participants were recruited between February 2022 and December 2023, and 40 commenced monitoring. Four did not commence monitoring and were excluded from analysis (Figure 2). The mean age of the participants was 48 years (SD = ±12) (range, 25–79 years; median age, 49 years). The majority of them were female (63.6%, *n* = 28). Socioeconomic factors (income, education, occupation, and social support) are deemed to influence access to technology, including the ability to afford smartphones or technology-based solutions (telehealth/wearable devices), Internet connectivity, and digital health literacy, eventually leading to poorer healthcare outcomes in the individual (34). In our study, all the potential and eligible participants had smartphones and access to the Internet. Those requiring assistance were guided via teleconsultation on how to install the S-Patch app onto their mobile phones, use the device, and check their reading on the phone. Socioeconomic factors did not influence the accessibility of the intervention.

#### 3.1.1 Data summary

Out of 40 patients who commenced monitoring, 13 patients (32.5%) were detected by the AI algorithms from the S-Patch EX monitoring system to have cardiac arrhythmias, including AF, supraventricular tachycardia (SVT), and ventricular tachycardia (VT) (Patients 3, 6, 7, 8, 10, 12, 18, 28, 30, 35, 37, 40, 42). Moreover, 77% (*n* = 10) of them were female. Ten of them had pre-existing comorbidities (10/13), with hypertension, Type 2 diabetes, and hypercholesterolaemia as the most common (Supplementary Table S1).

Arrhythmia was detected within a 3-month timeframe in most of the remote monitoring patients (Supplementary Table S1). As soon as cardiac arrhythmia was identified and confirmed by the participants, GP, or treating specialist, the participants were separated from the study.

##### 3.1.1.1 Mean of QRS complexes, supraventricular ectopy (SVE)%, and ventricular ectopy (VE)% for all patients

**Figure 3:**
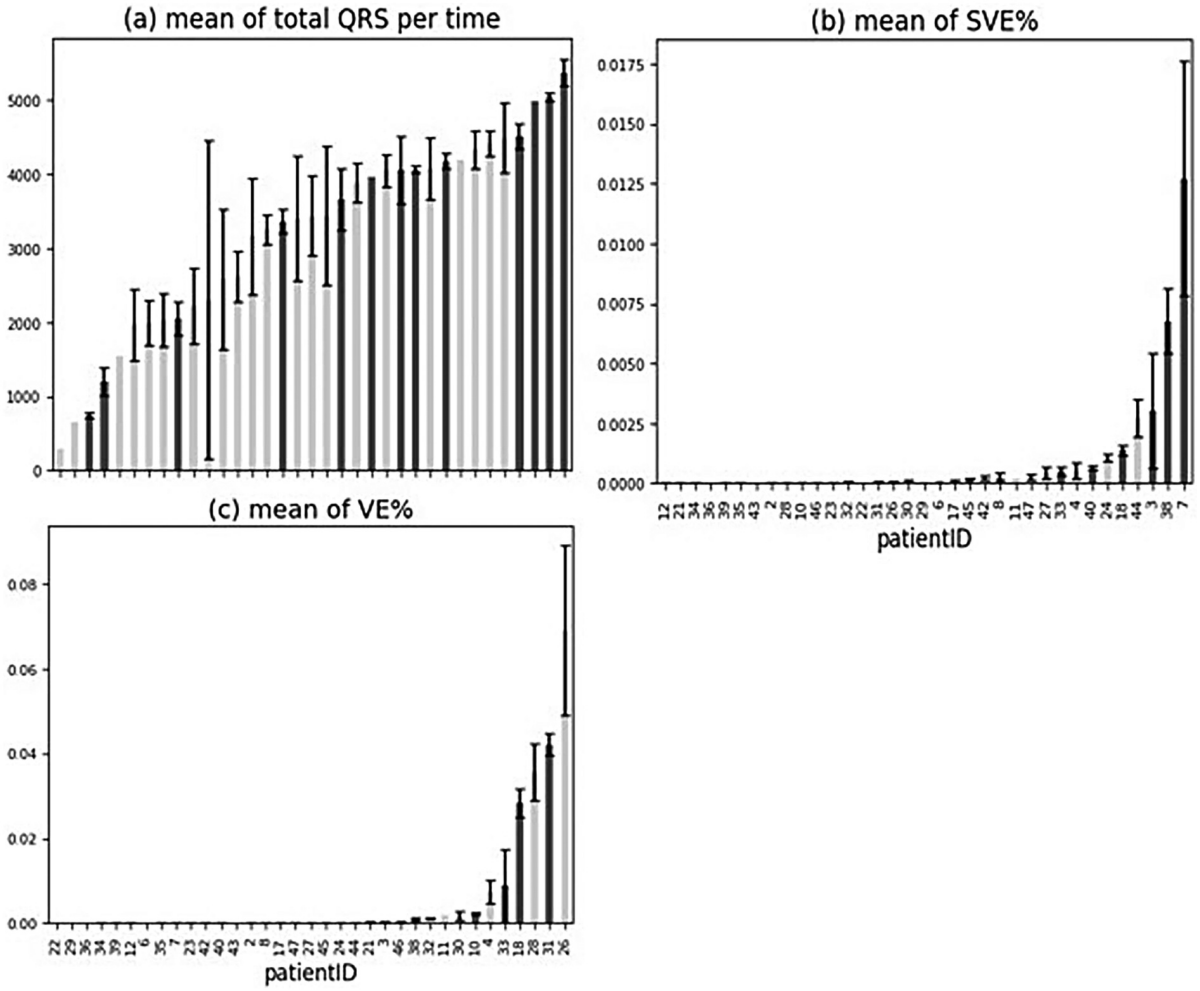
Mean of ORS complexes, SVE%, and VE% for all patients. **(a–c)** Patients with arrhythmia are shown in dark grey. Error bars indicate ± standard error of the mean.

The percentage (%) of total QRS complexes in relation to the total recording time per patient was analysed (Figure 3a), and it did not show any trend between the number of QRS complexes and cardiac arrhythmia occurrence. The average of SVE% (the ratio of SVE to the number of QRS) by patients was observed, and the patients with arrhythmias had a higher occurrence of SVE on the tests they performed (Figure 3b). The means of SVE% for the patients (especially Patients 18, 3, 38, 7) tended to be higher than for the other patients (Figure 2c). In summary, the % occurrence of SVE per test might be correlated to cardiac arrhythmias. In contrast to SVE, the average of VE% (the ratio of VE to the number of QRS) was not correlated to cardiac arrhythmias since no trend was observed (Figure 3c).

##### 3.1.1.2 Change of QRS complexes, SVE%, and VE% across tests within arrhythmia patients

**Figure 4:**
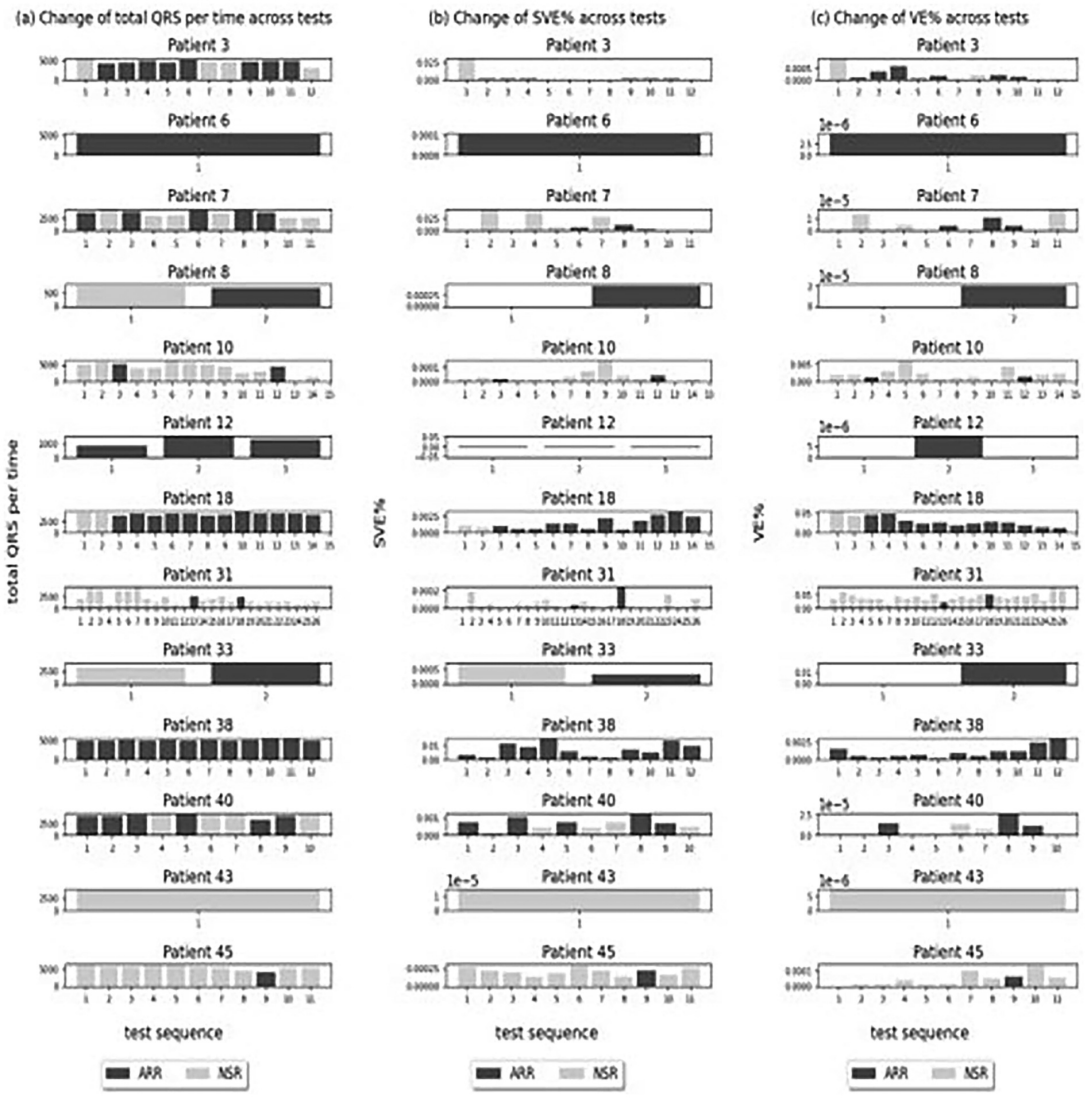
Change of QRS complexes, SVE%, and VE% across tests within arrhythmia patients. **(a–c)** Tests with arrhythmia detected.

After examining the overall trend across all patients and investigating the change of these statistics across tests within the patients with cardiac arrhythmias (Patients 3, 6, 7, 8, 10, 12, 18, 31, 33, 38, 40, 43, 45), we found no trend between the number of QRS complexes and arrhythmia occurrence throughout the tests. They did not increase or decrease across the tests, and the test with arrhythmias did not show particularly high or low numbers of QRS complexes (Figure 4). The change of SVE% (the ratio of SVE to the number of QRS) and VE% (the ratio of VE to the number of QRS) was also analysed, and no trend was found in relation to the arrhythmias across tests. They did not increase or decrease across the tests, and the test with arrhythmias did not show particularly high or low SVE% and VE%.

### 3.2 Standard care group (control)

The standard care group comprised 220 adults (5:1 ratio) with COVID who did not subsequently receive the study interventions. Twenty patients who had heart issues prior to COVID infection were excluded from the data analysis. The mean age was 46 years (SD = 12.7) (range, 25–79 years; median age, 44 years), and 60% of them were female. Arrhythmia was reported in 3.5% (7/200) of patients (supraventricular tachycardia, 5; bradycardia, 2), and 3 patients reported other cardiovascular conditions (coronary artery disease, 1; aortic aneurysm, 1; congestive cardiac failure, 1) (Supplementary Table S2). Twenty-one patients (10.5%) visited ED or were admitted to the hospital post-COVID infection due to chest pain, shortness of breath/dyspnoea, palpitations, dizziness/light-headedness/presyncope, and nausea (chest pain, 11; palpitations, 6; shortness of breath/dyspnoea, 8; dizziness/light-headedness/presyncope, 6; and nausea, 2). Two patients developed long COVID symptoms (progressive dyspnoea) 2–5 months post-COVID infection.

### 3.3 Remote monitoring group vs. standard care group

Table 1 shows the comparison of age, gender, and comorbidities by group. There were no significant differences in age and comorbidities; however, there was a significantly higher proportion of females in the remote monitoring group (25.2% females vs. 9.4% males).

**Table 1:** Clinical profile of the remote monitoring group (intervention) and standard care group.

Variables	Remote monitoring (*N* = 40)	Standard care (*N* = 200)	*P*-value
Age (mean ± SD)	48.19 ± 12.32	45.78 ± 12.68	0.2525
Gender, *n* (%)			
Female	27 (67.5%)	80 (60.9%)	0.4339
Male	13 (32.5%)	120 (39.1%)	
Comorbidities, *n* (%)			
Yes	30 (70%)	132 (68%)	0.2673
No	10 (30%)	68 (34%)	

Univariate Cox regression demonstrates that an arrhythmia was more likely to be detected in the remote monitoring group as compared with the standard care group [HR = 33.59, 95% CI (10.4, 108.47), *p* < 0.001] (Table 2). Increased age was also associated with detection of arrhythmia [HR = 1.05, 95% CI (1.01, 1.08), *p* = 0.008]. Gender and comorbidities were not associated with detection of arrhythmia. In multivariable analysis, arrhythmia was more likely to be detected in the remote monitoring group [HR = 31.1, 95% CI (9.61, 100.5)] after accounting for age.

**Table 2:** Cox regression models for time to cardiovascular event (arrhythmia).

Variables	Univariate			Multivariable		
	Hazard ratio	95% CI	*P*-value	Hazard ratio	95% CI	*P*-value
Group						
Remote monitoring	33.59	(10.4, 108.5)	<0.001	31.1	(9.6, 100.5)	<0.001
Standard care	Reference			Reference		
Age	1.05	(1.01, 1.08)	0.008	1.04	(1.01, 1.08)	0.024
Gender						
Female	1.04	(0.41, 2.65)	0.9297			
Male	Reference					
Comorbidities						
Yes	0.83	(0.42, 2.92)	0.832			
No	Reference					

## 4 Discussion

This study aimed to co-design and implement an innovative, novel model of virtual care, in a primary healthcare setting using advanced technologies and AI (S-Patch EX) to remotely monitor and identify clinical signs and symptoms of cardiovascular conditions (mainly arrhythmias) post-COVID infection and further assess rate of cardiac arrhythmia detection in remote monitoring group compared with the standard care group. Our study findings demonstrate that arrhythmia is more likely to be detected in the remote monitoring group as compared with the standard care group. The continuous data from S-Patch EX and its AI technology enabled further investigation into the changes in cardiac physiology for the study participants.

A number of studies have reported increased risk and burden of cardiac arrhythmia in patients post-COVID with a follow-up period ranging from 3 to 18 months (3, 4, 35–42). In our study, arrhythmia was detected in 32.5% of the participants in the remote monitoring group and 3.5% in the standard care group. Arrhythmia was detected within a 3-month timeframe in most of the remote monitoring patients. Similarly, in a study by Ingul et al. (35), cardiac arrhythmias were detected in 27% of the COVID patients at 3 months post-hospital discharge. Cases showed mildly impaired right ventricular function and higher rates of ventricular arrhythmias compared with the control group. In a study from China, with follow-up at different time frames (3, 6, and 12 months), arrhythmia was present in 12.4% patients at 3 months, 7.6% at 6 months, and 16.3% at 12 months post-discharge (36). Another study with a long follow-up period of 18 months identified arrhythmia in 10.4% of patients; however, the proportion was low compared with that of our study (37). Unlike these studies, in a multicentre study, no significant overall change in measures of cardiac structure and function from 3 to 12 months after COVID was observed. Among patients with arrhythmia, there was no significant change in arrhythmia burden at 3 to 12 months (43).

Follow-up studies have reported cardiopulmonary symptoms such as fatigue, dyspnoea, palpitations, shortness of breath, chest pain or pressure, and dizziness, presyncope, or syncope persisting in patients up to 2 years or more after acute infection (44, 45). In our study, 21 patients (10.5%) from the standard care group visited the ED or were admitted to the hospital post-COVID infection due to chest pain, shortness of breath/dyspnoea, palpitations, dizziness/light-headedness/presyncope, and nausea. Two patients developed long COVID symptoms (progressive dyspnoea) 2–5 months post-COVID infection. Studies suggest that patients with long COVID often suffer from CVD symptoms; however, some may not have an underlying CVD pathology.

Association between advancing age and arrhythmia is eminent in the literature (46). With increasing age heart and blood vessels undergo changes that can increase the risk of arrhythmias. Likewise, in our study, increased age was associated with detection of arrhythmia [HR = 1.05 (1.01, 1.08), *p* = 0.008].

Patients with pre-existing CVD are reported to have a significantly higher risk of developing cardiac-related long COVID symptoms (43, 47, 48). However, new-onset cardiac conditions have also been demonstrated in individuals without any previous history of CVD (3, 47, 49, 50). Similarly, in our study, arrhythmia was reported in patients who had no previous history of CVD (in the remote monitoring group as well as in the standard care group). Nevertheless, cardiovascular changes have also been reported in young, healthy asymptomatic or mildly symptomatic individuals (51, 52) or who seem to be completely recovered from the acute phase of infection (53). These findings highlight the need for continuous monitoring and evaluation as a part of post-COVID management for all patients, including young, healthy, and asymptomatic populations.

Remote digital monitoring has emerged as a growing and necessary aspect of COVID management for patients with cardiac complications or at risk of cardiac complications. Studies show that telemedicine solutions are useful and cost-effective methods, benefiting patients, carers, and doctors, as they allow identification and assessment of a patient's health remotely, alerting about any parameter changes, and enabling earlier detection and management (21, 54). However, the implementation of these telemedicine solutions requires engagement of a multidisciplinary team, who can monitor these vital signs on a regular and ongoing basis [including communication and assessment of the information and escalation (if required)]. We achieved this in our study conducted to co-design and deploy an innovative virtual model of care to fill the current gap, leveraging advancements in technologies to expedite the development and implementation of a digital intelligence model of cardiac health screening and monitoring. The project team also developed and utilised a communications platform to connect all the healthcare professionals in the cardiac care pathway to ensure the patients received early screening via a virtual, in-home healthcare programme and activate appropriate interventions if required.

A recent qualitative study from Australia highlights the challenges faced by general practice and practitioners in the diagnosis and management of long COVID (55). Primary healthcare providers may require more guidance on handling patients with long COVID and education on how to diagnose long COVID and best support those with the condition by means of easy-to-apply guidelines and screening tools relevant to the Australian context (55). Our study will support the primary healthcare providers and improve:

• Patient outcomes through earlier detection of deteriorating cardiac health, enabling timely treatment to prevent stroke or heart failure.
• Health practices and efficiency by embedding advanced technologies and AI into clinical operations to provide appropriate care in the community.
• Efficiency in the health system by developing simple, automated, remote patient monitoring, directing services to those in greatest need, and preventing avoidable hospital readmission.
• Community satisfaction by making in-home health services more accessible, affordable, and effective, reducing the burden of healthcare.
• Our healthcare model could subsequently be extended to rural and remote areas and will contribute to global understanding of the long-term sequelae of COVID.

### 4.1 Implications for policy and communications

Through the co-developed and implementation of a safe, timely, and cost-effective novel digital telehealth for cardiac health and communication platform, our study is positioned at the very forefront of delivering a standardised, evidence-informed, quality, and safe model of care at local, national, and international levels. This model of care allows patients to remotely monitor their ECG at home and transmit the ECG data in real-time to the S-Patch portal for initial analysis and reporting by an AI algorithm, followed by review from a clinician, without hospital visits. It also improves patients' access to early screening and facilitation of early diagnosis and intervention from their primary healthcare providers.

### 4.2 Limitations of the study

During the study period, the majority of the people in Sydney were vaccinated. They received either one dose or a double dose, and some even received booster doses. Emerging evidence suggests that COVID vaccination is associated with a lower risk of persistent post-COVID symptoms (56) and reduced major adverse CVD/ cerebrovascular and thromboembolic complications (41, 57). In our study, we did not collect data on vaccination status. The vaccination status could have impacted the prevalence of the reported arrhythmias.

One of the limitations was the open-label non-randomised study design. Considering the difficulty with recruiting patients in the intervention group and the cost associated with the intervention, we opted for this study design. However, we sought to mitigate the bias and confounding through vigilant study design (well-defined and clear inclusion/exclusion criteria with similar baseline confounders, utilising appropriate control groups) and statistical analysis (using a regression model).

We also acknowledged that in the standard care group, we only assessed data available in the hospital records. There was a possibility that some patients might have visited and sought treatment from a different setting (other hospital or general practitioner), and this could have led to underdetection of arrhythmia in the standard care group.

### 4.3 Future study

Patients reporting cardiopulmonary symptoms post-COVID should be subjected to basic CVD evaluation to detect arrhythmias. Stepwise screening can potentially identify patients with CVD (58–60). The study involved a small cohort. A large randomised interventional study including patients from the long COVID clinics and accounting for factors such as vaccination status with a longer follow-up period would be advised for a better understanding of cardiovascular impact and to further implement the advanced in-home technology and data integration to inform a quality, safe, timely, effective, and structure cardiac care pathway. Economic evaluation and ethical and user considerations would also be recommended.

## 5 Conclusion

Our findings demonstrate that arrhythmia was more likely to be detected in the remote monitoring group as compared with the standard care group. Given the risk of developing cardiovascular complications in patients with COVID, during and post-infection, regular monitoring and reassessment are recommended. Healthcare providers should incorporate monitoring and reassessment as a part of post-COVID management for all patients.

## Data Availability

The raw data supporting the conclusions of this article will be made available by the authors, without undue reservation.
