# Supplementary material for: Remote, smart telemonitoring of COVID-19 survivors for early detection of deterioration in cardiac health (the PARTMO study)
Source: Front Med Technol. 2025 Jul 24;7:1534097. doi: 10.3389/fmedt.2025.1534097 (PMC12328397; doi:10.3389/fmedt.2025.1534097)
Supplement: Supplementary file 1 [file Table1.docx]

**Supplementary Tables**

**Table 1: Demographic and clinical profile of Remote Monitoring Group (Arrhythmia detected during monitoring)**

| **Patient ID** | **Date of Admission** | **Gender** | **Age (years)** | **Hospitalization status (Yes/No)** | **Existing comorbidities** | **Date started monitoring** | **Date of 1st event**  **(Arrhythmia detected)** |
| --- | --- | --- | --- | --- | --- | --- | --- |
| P-3 | 9/4/2021 | F | 56 | Yes | T2DM, Asthma, Hypercholesterolaemia, HTN, hypothyroidism | 23/02/2022 | 11/05/2022 |
| P-6 | 14/7/2021 | M | 43 | Yes | HTN, T2DM, Hypercholesterolaemia | 21/02/2022 | 23/02/2022 |
| P-7 | 28/10/2021 | F | 53 | Yes | Iron deficiency | 09/03/2022 | 29/05/2022 |
| P-8 | 16/11/2021 | M | 49 | Yes | Hyperglycaemia, Iron Deficiency | 08/03/2022 | 10/04/2022 |
| P-10 | 24/7/2021 | F | 66 | Yes | Asthma, HTN, Iron deficiency anaemia, Obesity | 10/03/2022 | 1/05/2022 |
| P-12 | 6/9/2021 | F | 51 | Yes | Asthma, HTN, High cholesterol, Obesity | 08/03/2022 | 16/03/2022 |
| P-18 | 23/11/2021 | M | 79 | Yes | HTN, T2DM, Hyperlipidaemia | 23/03/2022 | 4/05/2022 |
| P-28 | 24/7/2021 | F | 51 | Yes | T2DM, High Cholesterol | 31/05/2023 | 10/10/2023 |
| P-30 | 13/07/2022 | F | 54 | No | Nil | 17/07/2023 | 31/10/2023 |
| P-35 | 2/05/2022 | F | 56 | No | HTN | 07/08/2023 | 11/12/2023 |
| P-37 | 1/02/2022 | F | 65 | No | Nil | 04/08/2023 | 11/11/2023 |
| P-40 | 12/04/2022 | F | 48 | No | Nil | 18/09/2023 | 28/09/2023 |
| P-42 | 1/07/2022 | F | 50 | No | HTN, GDM | 28/08/2023 | 30/11/2023 |

HTN – Hypertension, T2DM - Type 2 diabetes mellitus, PTSD - Post-traumatic stress disorder, COPD - Chronic obstructive pulmonary disease, GDM - gestational diabetes mellitus, IHD - Ischemic heart disease, AF - Atrial fibrillation, DVT - deep vein thrombosis

**Table 2: Demographic and clinical profile of Standard Care group (with CVD)**

| **Patient ID** | **GENDER** | **Age in Years** | **Admission date** | **Medical History (existing comorbidities)** | **Hospitalization/ED visit due to heart issues post COVID** |
| --- | --- | --- | --- | --- | --- |
| C2 | M | 76 | 25/08/2021 | Depression, Hypercholesterolaemia, BPH, GORD, COPD, Diverticular disease | Aortic Aneurysm (22/8/2022), Chest pain (1/9/2023) |
| C44 | M | 51 | 22/02/2022 | HTN | Chest pain, SOB Hypertensive and tachycardic (5/03/2023) |
| C75 | F | 36 | 17/03/2022 | Nil | (07/02/2023) presented with presyncope episodes. ECG - Mild Tachycardia |
| C127 | F | 55 | 23/08/2021 | Asthma, GORD, HTN, Hyperlipidaemia | LAD Coronary Disease (1/9/2022), Chest Heaviness (24/5/2023), Chest Pain (10/7/2023) |
| C128 | M | 62 | 21/07/2021 | HTN, ex-smoker, Hyperlipidaemia | ED visit with Supraventricular tachycardia (11/01/2024) |
| C144 | M | 32 | 25/05/2021 | Depression, severe TBI, Hypopituitarism, Tracheostomy | Hypotension, Bradycardia (25/8/2022) |
| C166 | F | 74 | 04/11/2021 | Kidney cancer with R nephrectomy, reflux oesophagitis, HTN, T2DM | Bradycardia (10/11/2023) |
| C171 | F | 64 | 11/03/2022 | Cellulitis, HTN, OSA, T2DM | (06/03/23) Congestive cardiac failure. (17/03/23) exacerbation of CCF. |
| C188 | M | 56 | 22/01/2022 | Nil | (30/06/2024) Presented to ED with palpitations and presyncope and hypotension (SBF-72). ECG - showing SVT. |
| C191 | F | 54 | 29/07/2021 | Nil | HR slightly tachycardiac (17/5/2024) |

HTN – Hypertension, T2DM - Type 2 diabetes mellitus, GDM - gestational diabetes mellitus, NA- Not Applicable
